# Usefulness of sepsis screening tools and education in recognising the burden of sepsis on hospital wards

**DOI:** 10.1186/cc14083

**Published:** 2015-03-16

**Authors:** EJ Galtrey, C Moss, H Cahill

**Affiliations:** 1 Guy's and St Thomas' Hospitals NHS Foundation Trust, London, UK

## Introduction

Sepsis is defined as the presence of infection with systemic signs of infection, and severe sepsis as sepsis plus sepsis-induced organ dysfunction or tissue hypoperfusion [1]. Since the Surviving Sepsis Campaign (SSC) in 2002, the Health Service Ombudsman for England published recommendations for improving recognition and treatment of sepsis [2], the Royal College of Physicians issued a toolkit for the management of sepsis in the acute medical unit [3], and NHS England released a patient safety alert to support prompt recognition and treatment of sepsis [4]. In 2012 our Trust introduced a sepsis screening tool and electronic order set (EPR alert) alongside an education programme to improve delivery of the SSC bundle. Previous audits showed only 43% full bundle compliance in those that were alerted, and this raised concerns regarding the burden of unalerted sepsis. We sought to estimate the number of unalerted sepsis episodes to assess the efficacy of our screening tool and improve early recognition.

## Methods

All referrals to our critical care response team with a diagnosis of sepsis over a 3-month period (September to November 2014) were investigated to determine how many had an EPR sepsis alert comprising a prompt for blood cultures, serum lactate measurement, fluid challenge if hypotensive, and antibiotics within 1 hour.

## Results

Only 25/174 (14%) patients with a diagnosis of sepsis had an EPR sepsis alert. There was no significant difference between acute and nonacute ward areas in their likelihood of using the screening tool or alert, in contrast to previous audits of the alerted population which showed that acute areas such as A&E and medical acute admission wards had higher utilisation and bundle completion rates.

## Conclusion

Despite these interventions, most patients still do not receive the full recommended treatment bundle. These findings have prompted a point prevalence audit at ward level, which will examine all patients' notes for the preceding 24 hours to ascertain if sepsis is truly unrecognised or whether it is simply that our current tool is not a helpful adjunct to care. With national guidelines expected within the year, we will redesign and re-launch our screening tools and education programme to improve awareness and management of this common medical emergency.
